# Guillain–Barre Syndrome With Cytomegalovirus Infection After Allogeneic Hematopoietic Stem Cell Transplantation

**DOI:** 10.1002/kjm2.70225

**Published:** 2026-04-23

**Authors:** Chin‐Mu Hsu, Ping‐Hsuan Lin, Chia‐Hsuan Chiu, Hui‐Hua Hsiao

**Affiliations:** ^1^ Division of Hematology‐Oncology, Department of Internal Medicine Kaohsiung Medical University Hospital Kaohsiung Taiwan; ^2^ Cancer Center, Kaohsiung Medical University Hospital Kaohsiung Taiwan; ^3^ Faculty of Medicine Kaohsiung Medical University Kaohsiung Taiwan

Dear Editor,

1

Guillain–Barre syndrome (GBS) is an acute inflammatory polyneuropathy characterized by rapid, progressive ascending and symmetrical weakness and areflexia [1]. It is a rare neurological complication after hematopoietic stem cell transplant (HSCT), which is typically triggered by cytotoxic agents, GVHD, and, rarely, antecedent infections [2]. Herein we report a transplant patient who suffered from GBS after CMV infection.

A 47‐year‐old male patient suffered from acute myeloid leukemia with IDH1 mutation in January, 2025. He received Idarubicin and Cytarabine (3 + 7), and decitabine plus venetoclax therapy with response. Due to high‐risk profile, he underwent allogeneic hematopoietic stem cell transplant from a full match sibling donor in September, 2025 with complete remission and full donor chimerism. However, CMV pneumonia with high CMV viral load was noted on day 58 after transplantation, then the infection was brought under control with anti‐viral agent. He was discharged from the hospital at a later time. Unfortunately, rapid, ascending, and progressive weakness from legs to arms occurred on Day 92. Readmission and further NCV testing revealed polyneuropathy over both sensory and motor nerves, although CSF study revealed no cells with high IgG in CSF. At the same time, CMV viral load relapsed again, and under the impression of GBS associated with CMV infection, steroids with plasma exchange were administered and the symptoms improved; however, a flareup of symptoms was found after discontinuation of 10 cycles of plasma exchange leading to progression of dysphagia and progressive weakness. Subsequently, the patient received further courses of plasma exchange without worsening of the symptoms, and was eventually discharged from the hospital after clinical stabilization.

Peripheral neuropathies after stem cell transplantation are uncommon and generally result from cytotoxic agents and/or GVHD after HSCT. GBS developing from an immunologically mediated reaction from infection has been reported only in a small subset of patients after HSCT with poor prognosis [2, 3], with antecedent CMV infection before GBS being rarely observed. Molecular mimicry between microbial antigens and host tissue has been postulated as the triggering mechanism of this immune reaction [4]. Anti‐viral therapy and therapeutic plasma exchange are the main treatment options; however, novel agents are warranted to improve the outcomes [5]. In conclusion, GBS is a rare but serious complication after HSCT. Increased awareness for early detection and early intervention is crucial in the management of GBS.

## Conflicts of Interest

The authors declare no conflicts of interest.

## Data Availability

The data that support the findings of this study are available from the corresponding author upon reasonable request.
